# SLMAP3 is crucial for organogenesis through mechanisms involving primary cilia formation

**DOI:** 10.1098/rsob.240206

**Published:** 2024-10-17

**Authors:** Ana Paula Dias, Taha Rehmani, Billi Dawn Applin, Maysoon Salih, Balwant Tuana

**Affiliations:** ^1^ Department of Cellular and Molecular Medicine, University of Ottawa, Ottawa K1H 8M5, Canada

**Keywords:** SLMAP3, STRIPAK, FGFR1OP2, PCP, primary cilia, Hippo pathway

## Abstract

SLMAP3 is a constituent of the centrosome and is known to assemble with the striatin-interacting phosphatase and kinase (STRIPAK) complex, where it has been reported to repress Hippo signalling. The global knockout of SLMAP3 in mice results in embryonic/perinatal lethality and stunted growth without changes in the phosphorylation status of YAP. Diverse phenotypes present in the SLMAP3^−/−^ embryos include reduced body axis, small and abnormal organs resembling defects in planar cell polarity (PCP) signalling, while also displaying the notable polycystic kidneys, a known manifestation of ciliopathies. Analysis of cell polarity in primary mouse embryonic fibroblasts (MEFs) including cell migration, orientation and mitotic spindle angle did not reveal any changes due to SLMAP3 loss in these cells, although the expression of DVL3 was significantly reduced. Furthermore, MEFs lacking FGFR1OP2 or STRN3, two other STRIPAK members, did not reveal any significant changes in any of these parameters either. Significant changes in the number of ciliated cells and primary cilium length in SLMAP3 and FGFR1OP2 deficient MEFs were evident, while a reduced primary cilium length was notable in chondrocytes of SLMAP3 deficient embryos. Our findings suggest that SLMAP3 is essential for mouse embryogenesis through novel mechanisms involving the primary cilium/PCP and protein stability independent of Hippo signalling.

## 1. Introduction

Congenital disorders are pathological conditions that stem from functional or structural defects, causing an estimated death of 295 000 infants worldwide every year and are one of the leading causes of death of people under 5 years old [1]. With 50% of these disorders without a specific cause [1], studies investigating birth defects can contribute to prevention strategies, treatment and early diagnosis.

Here, we investigated the impact of SLMAP3 deficiency on mouse embryonic development. The slmap gene encodes multiple protein isoforms that are expressed in tissue-specific manner generated through alternative splicing [2–4]. SLMAP proteins have in common coiled-coil domains, which favour protein–protein interactions, and C-terminal transmembrane domains that can target the protein to subcellular organelles, including endoplasmic reticulum, sarcolemma, mitochondria and nuclear envelope [2–6]. SLMAP3, which is ubiquitously expressed, encompasses the largest isoform that additionally has a N-terminal forkhead-associated (FHA) domain [4], known to bind to phosphothreonine residues [7,8] and to target SLMAP to the centrosome [4,9]. We reported that the deficiency of SLMAP3 in skeletal myoblasts impairs the microtubule organizing centre formation (MTOC) in the nuclear envelope leading to defective myogenesis [6]. SLMAP has also been associated with Brugada syndrome, a fatal cardiomyopathy due to defective ion channel trafficking [10].

SLMAP3 and its paralogue TRAF3IP3 are constituents of the striatin-interacting phosphatase and kinase (STRIPAK) complex [11,12], composed of the phosphatase PP2A, kinases from the germinal centre kinases families II, III and IV, the adaptors paralogues STRIP1 and STRIP2, Mob4, the paralogues STRN, STRN3 and STRN4, CCM3, the paralogues CTTNBP2 and CTTNBP2NL and the paralogues SIKE1 and FGFR1OP2 [11–17]. STRIPAK is evolutionarily conserved, being described in fungi [18], in invertebrates such as *Drosophila melanogaster* [19–22]*, Caenorhabditis elegans* [23] and planarians [24], and in vertebrates [11,12]. The high conservation of SLMAP3 in the context of STRIPAK suggests that it might share essential roles in eukaryotes. The importance of STRIPAK in mammals is further evidenced by the deficiency of many of its components resulting in mouse embryonic lethality, as observed in the case of PP2A-C [25,26], PP2A-A [27,28], MAP4K4 [29], STRN [30], STRN3 [31,32], STRIP1 [33], Mob4 [34], CCM3 [35,36] and FGFR1OP2 [37]. The crystal structure of STRIPAK revealed that STRN3 proteins function as a scaffold, and STRIP1 and Mob4 help stabilize the complex [38]. SIKE1 interacts with STRN3 at an outer layer by which it recruits SLMAP3 to the complex [38,39]. FGFR1OP2 as a paralogue of SIKE1 could recruit SLMAP3 in a similar fashion although little is known about its role. FGFR1OP2 was described to enhance wound healing [40–42] and found fused with FGFR1 receptor in myelomas [43–45].

In the context of STRIPAK, SLMAP3 was shown to be a negative regulator of Hippo signalling by recruiting the germinal centre kinases II MST1/2 to the complex via its FHA domain, where these kinases are dephosphorylated and inactivated by the PP2A [13,14,20,46]. This ultimately reduces the phosphorylation of the transcriptional co-activators YAP/TAZ, allowing their translocation from the cytoplasm to the nucleus, where they induce the expression of genes for cellular proliferation and growth [47,48]. The isoforms SLMAP1/2 (Uniprot B7Z863 and B7Z964, respectively) do not participate in this signalling because they lack the FHA required for the interaction with MST1/2. Because the Hippo pathway studies were conducted in cultured cells, we investigated the impact of the global ablation of SLMAP3 *in vivo*. We used the cre-lox to engineer the global knockout of slmap in mice, which presented embryonic/perinatal lethality [6]. Here, we delved into detailed analysis of the phenotypes and potential mechanisms of SLMAP3 action including Hippo signalling. The phenotypes observed with SLMAP3 loss include overall stunted growth with underdeveloped lungs, fused skeleton, disorganized chondrocytes in the growth plate, short intestines, polycystic kidneys and short tails, deficits that are consistent with those reported for aberrant planar cell polarity (PCP) [49–63].

PCP is crucial for cell polarity by guiding the asymmetric distribution of proteins such as DVLs to govern the morphogenic process of convergent extension and organogenesis [64–68]. The primary cilium, a non-motile microtubule-based projection, which is present in virtually all cells of the body and is crucial for detecting environmental cues to dictate cell behaviour [69,70], has also been linked to PCP [71–81]. PCP has been described to influence the polarization of migratory cells [82–86] and the orientation of mitotic spindle [87–91], and the latter also regulated by the centrosomal protein pericentrin [92] and ciliary protein IFT20 [93]. We report that loss of SLMAP3 reduces the expression of the PCP protein DVL3 and impacts the primary cilium without any effect on Hippo signalling. Given that SLMAP3 is a centrosomal component, our results point towards an entirely new mode of action for this protein through mechanisms involving primary cilium and PCP components.

## 2. Results

### 2.1. SLMAP3^−/−^ mice display embryonic/perinatal lethality with defective tissue architecture

**Figure 1.**
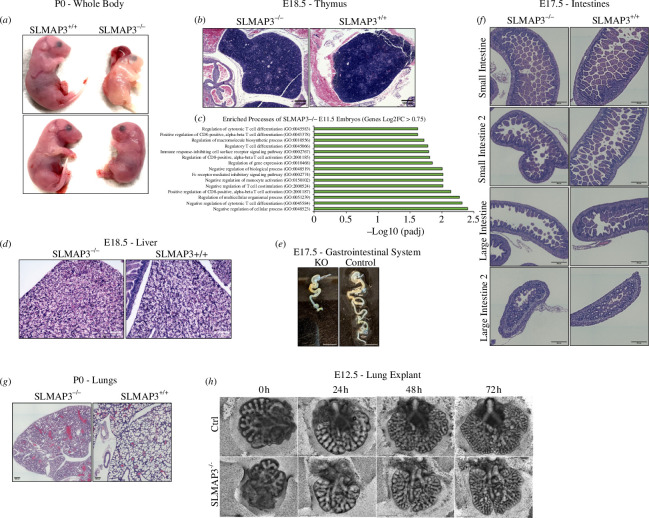
SLMAP3^−/−^ mice display organ deficits and embryonic/perinatal lethality. (*a*) Knockout of SLMAP3 leads to embryonic/perinatal lethality, with animals presenting smaller limbs and tails, reduced body size and cases of craniorachischisis. (*b*) Thymus of animals lacking SLMAP3 shows reduced cell density, (*c*) which is consistent with the enrichment of biological processes associated with T-cell differentiation/activation. For the gene ontology enrichment analysis, we used the G:Profiler tool [94] with genes with significantly altered expression and log2 fold change > 0.75 comparing SLMAP3^−/−^ and WT, from data obtained in our previous RNA-seq with E11.5 embryos [6]. (*d*) The histology of the liver does not indicate major structural defects, although the organs are smaller in SLMAP3^−/−^ embryos. Scale bar = 100 μm. (*e*) Intestines of SLMAP3^−/−^ mice are shorter (scale bar = 5 mm), but (*f*) the regions of small and large intestines are properly specified. Scale bar = 200 μm. (*g*) Lungs of animals lacking SLMAP3 are underdeveloped with the absence of air sacs. Scale bar = 200 μm. (*h*) E12.5 lung explant of SLMAP3^−/−^ mouse displays branching morphogenesis comparable to control. Scale bar = 650 μm.

Previously, we have generated global SLMAP3^−/−^ mice using the Cre-Lox system [6]. The lack of SLMAP3 results in embryonic/perinatal lethality with 100% penetrance, and smaller embryos with small limbs and tails, abnormal face morphology and some embryos exhibit severe case of open neural tube known as craniorachischisis (figure 1*a*). The thymus of these animals are formed, with a notable reduced cell density (figure 1*b*). Furthermore, using our previous RNA-seq from E11.5 embryos [6] for gene ontology enrichment analysis with G:Profiler [94], we found that significantly altered genes with log_2_ fold change > 0.75 in SLMAP3^−/−^ embryos enrich for T-cell differentiation/activation (figure 1*c*), supporting the finding of abnormal thymus.

Although smaller, the liver architecture of mice lacking SLMAP3 does not seem to be affected (figure 1*d*). The intestines have reduced length (figure 1*e*), but the different regions of small and large intestines are properly specified (figure 1*f*). The lungs are underdeveloped, displaying thick alveolar epithelium with deficit in the formation of alveolar sacs, although terminal bronchioles and alveolar ducts are observable (figure 1*g*). The pulmonary defects may explain the respiratory distress and cyanosis observed in SLMAP3^−/−^ animals that are born alive but die soon after birth (electronic supplementary material, video S1). The changes in the lung development cannot be attributed to defective branching morphogenesis, as lung explants of embryos lacking SLMAP3 presented branch formation comparable to the controls after 72 h in culture (figure 1*h*).

**Figure 2.**
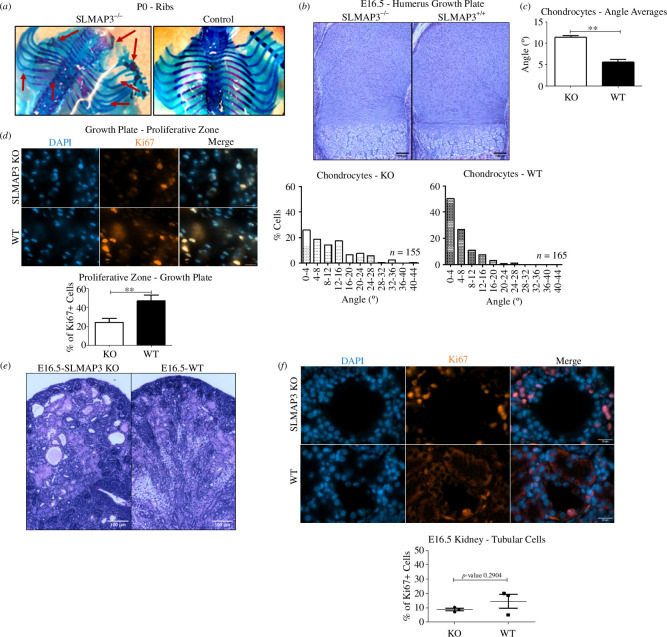
Loss of SLMAP3 results leads to structural deficits. (*a*) Alcian blue (cartilage) and alizarin red (bones) staining of SLMAP3^−/−^ mice reveals skeletal defects, with fused bones and cartilages of the ribs. (*b*) H&E staining of the humerus growth showing that chondrocytes lacking SLMAP3 have defective organization into columns and are disoriented, as indicated in the histograms. The number of cells considered for the histograms is indicated in the image and cells are from three different biological replicates. Scale bar = 100 μm. (*c*) The angle averages of the three biological replicates are plotted (*n* = 3). (*d*) The proliferative zone of the growth plate in SLMAP3^−/−^ mice has reduced Ki67+staining, suggesting reduced proliferation. Scale bar = 20 μm (*n* = 3). (*e*) The kidneys of SLMAP3^−/−^ mice are underdeveloped and polycystic. Scale bar = 100 μm. (*f*) Quantification of ki67+cells in the epithelium of proximal/distal tubules do not indicate increased proliferation in the cysts of SLMAP3^−/−^ mice (*n* = 3). Scale bar = 20 μm. **p < 0.01.

Additional noticeable phenotype observed in SLMAP3^−/−^ is the skeleton defects, with fused bones and cartilages (figure 2*a*). Examination of the humerus growth plate revealed defective organization of chondrocytes into columns (figure 2*b*), with defective orientation angle of the cells (figure 2*c*), which could explain the observed fused bones and cartilages. The proliferation of chondrocytes in the proliferative zone of the growth plate, assessed by Ki67 immunofluorescence staining, was found to be significantly reduced (figure 2*d*). Disoriented chondrocytes together with reduced proliferation could explain the defective limbs present in SLMAP3^−/−^ embryos.

The kidneys of SLMAP3^−/−^ mice are notably underdeveloped and present cysts (figure 2*e*). This type of polycystic kidney resembles those observed in the autosomal recessive disorder nephronophthisis, a typical infantile and bilateral disease where instead of massively large kidneys with cysts observed even in the surface of the organ, the kidneys can exhibit reduced size with cysts commonly located in the corticomedullary junction [95]. To investigate whether the cysts were formed due to uncontrolled proliferation, as it is seen in autosomal dominant polycystic kidney disease with mutations PKD1 and PKD2 genes [96], we assessed Ki67 staining in the epithelial cells of proximal and distal tubules and found no significant changes (figure 2*f*). Therefore, the cyst formation observed in SLMAP3^−/−^ is not attributable to increased cellular proliferation.

### 2.2. SLMAP3 functions independently of Hippo signalling during mouse embryogenesis

**Figure 3.**
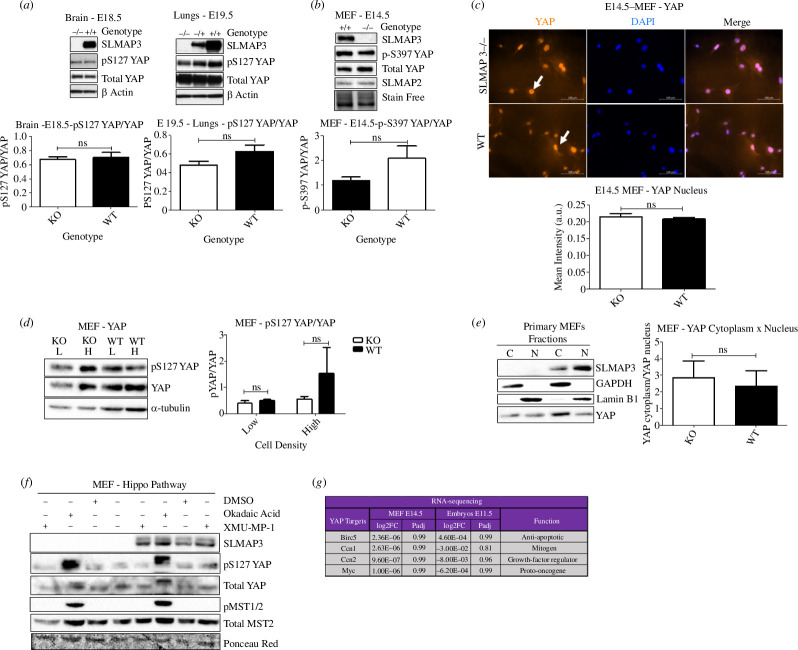
SLMAP3 acts independently of Hippo signalling in embryogenesis. (*a*) Loss of SLMAP3 does not affect the phosphorylation status of YAP in brain, lungs and (*b*) MEFs (*n* = 3). (*c*) Immunofluorescence for YAP/TAZ reveals their preferably nuclear localization in both SLMAP3^−/−^ and WT cells. Scale bar = 100 μm (*n* = 3) (*d*) Phosphorylation of YAP in SLMAP3^−/−^ MEFs in low and high cell density is similar to what is observed in WT cells (*n* = 3). (*e*) Analysis of YAP in both nuclear and cytoplasmic fraction does not indicate any enrichment of the protein in the cytoplasm of SLMAP3^−/−^ MEFs (*n* = 3). (*f*) Phosphorylation of MST1/2 is only observed when MEFs are treated with okadaic acid, indicating that these kinases are kept inactive independently of SLMAP3 presence. (*g*) RNA-sequencing of both embryos [6] and MEFs lacking SLMAP3 do not show changes in the expression of YAP target genes. For RNA-seq from embryos, for SLMAP3^−/−^ (*n* = 3), and for WT (*n* = 4); for RNA-seq in MEFs (*n* = 4) for both SLMAP3^−/−^ and WT cells. ns, not significant.

Given the stunted development and widespread organ deficits of SLMAP3^−/−^ embryos and the association of SLMAP with Hippo signalling and cell proliferation, we interrogated any impact on the phosphorylation status of YAP. Western blot analysis of multiple organs, including brain and lungs, indicated that there was no change in status of phosphorylated YAP due to SLMAP3 loss (figure 3*a*). This is in accordance with no changes in YAP phosphorylation in whole embryos, limbs and hearts lacking SLMAP3 [6,97–99]. Furthermore, we isolated mouse embryonic fibroblast (MEFs) from E14.5 SLMAP3 null embryos, which also indicated no significant changes in phosphorylation of YAP at serine 397 compared with WT cells (figure 3*b*). The immunofluorescence staining of YAP indicated similar nuclear localization in both WT and SLMAP^−/−^ MEFs without any significant differences (figure 3*c*). We also analysed the phosphorylation of YAP at low cell densities (figure 3*d*) and compared the distribution of YAP in the nuclear and cytoplasmic fractions (figure 3*e*), but found no differences between SLMAP^-/-^ MEFs and WT. Treatment of cells with either XMU-MP-1, an inhibitor of MST1/2 kinases [100], or okadaic acid, a PP2A inhibitor [101,102], did not reveal any changes in the phosphorylation status of YAP and MST1/2 between SLMAP3^−/−^ and WT MEFs (figure 3*f*). MST1/2 were particularly active only in the presence of okadaic acid in both cell types (figure 3*f*), indicating that in culture conditions, these kinases are normally kept inactive by mechanisms independent of SLMAP3. Furthermore, RNA-sequencing analysis of the two MEF genotypes (electronic supplementary material, table S1) indicated no differences in the expression of the YAP target genes Birc5, CCN1, CCN2 and Myc [103], similar to what is observed in our previous RNA-seq from whole SLMAP3^−/−^ embryos [6] (figure 3*g*).

We further examined protein lysates of proliferating MEFs by reverse phase protein array (RPPA) assay [104], in a panel consisting of 407 antibodies. The resulting relative protein expression is available in electronic supplementary material, table S2, and the unsupervised clustering and heat maps of these expressions are found in the electronic supplementary material S2. These antibodies targeted proteins from a large variety of biological processes, including Hippo pathway, canonical Wnt signalling, glycolysis, autophagy, embryonic development, mTOR, DNA damage, cell survival, proliferation, apoptosis and others. Despite the broad panel of antibodies, no obvious changes in protein levels/phosphorylation could be identified in SLMAP3^−/−^ MEFs. RNA-seq analysis of proliferating MEFs (electronic supplementary material, table S1) indicated that only 11 genes were expressed differently (electronic supplementary material, figure S1A), most notably Arhgap36 and CDKN1A with Log2 fold change of −2.34 and −0.60, respectively. The data were validated by RT-qPCR, confirming the downregulation of Arhgap36 (electronic supplementary material, figure S1B) and CDKN1A (electronic supplementary material, figure S1C). Together, these results suggest that gene expression in proliferating MEFs were not broadly impacted by the lack of SLMAP3, despite the severity of the phenotypes observed in SLMAP3^−/−^ embryos.

**Figure 4.**
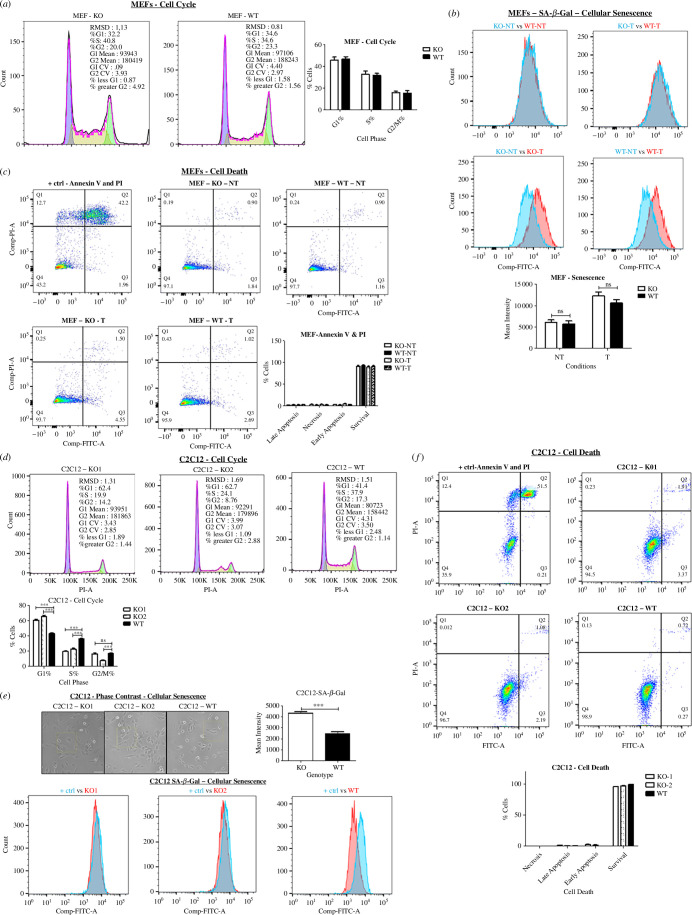
SLMAP3 impacts cell cycle dynammics in a cell-specific manner. (*a*) Cell cycle analysis by DNA staining with propidium iodide shows that knockout of SLMAP3 in MEFs does not cause any cell cycle arrest (*n* = 6), or (*b*) cellular senescence (*n* = 6), evaluated by SA-β-gal assay. (*c*) PI and Annexin V staining shows that the lack of SLMAP3 does not cause cell death in MEFs (*n* = 5). (*d*) Cell cycle analysis by DNA staining with propidium iodide indicates that both C2C12 KO cells have G1 cell cycle arrest (*n* = 3). (*e*) The phase contrast images of SLMAP3^−/−^ C2C12 cells show signs of cellular senescence: large, flat and vacuolated cells. Scale bar = 100 µm. This is consistent with their significantly higher β-galactosidase activity. For SLMA3 KO, *n* = 5, and for WT, *n* = 3. (*f*) PI and Annexin V staining do not suggest cellular death of both SLMAP3^−/−^ C2C12 myoblasts (*n* = 3). The positive controls for (*b*) and (*e*) consisted of WT cells treated with 600 µM of H_2_O_2_ to induce cellular senescence. For (*c*) and (*f*), the positive controls were treated with 1.6 mM H_2_O_2_ to induce cellular death, and the quadrants are Q1: necrosis; Q2: late apoptosis; Q3: early apoptosis; Q4: survival. For (*e*), the statistical analysis was performed by grouping the three biological samples of KO1 with two biological samples of KO2 and comparing to a group of WT with three biological replicates. ***p < 0.001; ns, not significant.

Cell cycle dynamics of SLMAP3^−/−^ MEFs with propidium iodide (PI) staining indicated no significant changes (figure 4*a*). Cellular senescence was not observed either, and with H_2_O_2_ treatment the senescence in SLMAP3^−/−^ MEFs was comparable to that in WT cells (figure 4*b*). Annexin V and PI staining analysis did not indicate changes in cell death, even after the induction of cellular stress with H_2_O_2_ (figure 4*c*). In comparison, we noted that myoblasts lacking SLMAP3 (electronic supplementary material, figure S2A) that we previously generated by CRISPR/Cas9 [6], resulted in accumulation of cells in G1 phase (figure 4*d*), followed by increased cellular senescence (figure 4*e*) without any changes in cell death (figure 4*f*). The increased cellular senescence in SLMAP3^−/−^ C2C12 myoblasts is in accordance with what we observed in H9C2 cardiomyoblasts with depletion of all SLMAP isoforms, which also exhibited reduced proliferation and increased senescence [98]. These findings suggest that SLMAP3 can influence cell cycle dynamics in a cell-specific manner and works through mechanisms that are independent of Hippo signalling.

### 2.3. Loss of SLMAP3 impacts cell shape independent of changes in cortical F-actin or focal adhesions

**Figure 5.**
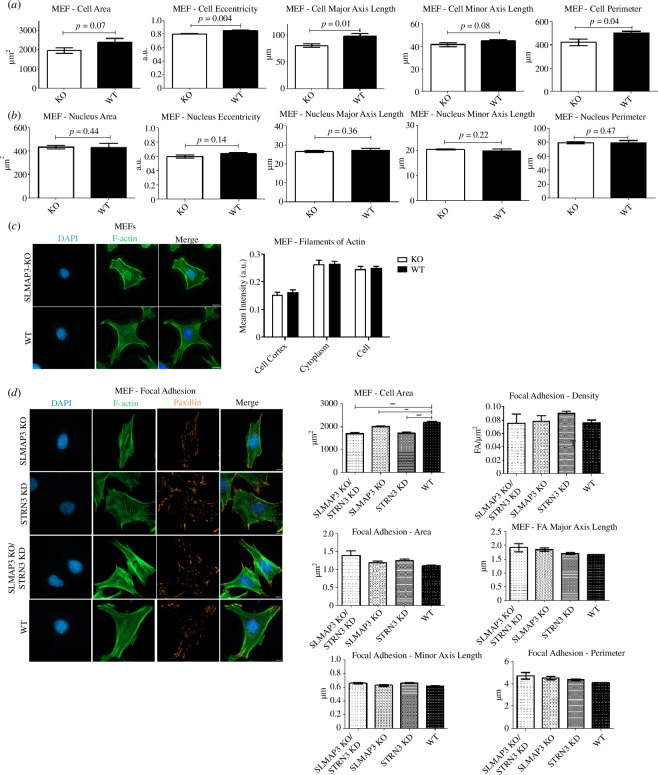
SLMAP3 impacts cell shape independent of cortical F-actin or focal adhesions. (*a*) MEFs lacking SLMAP3 display changes in cell shape parameters, while (*b*) no changes are observed in the nucleus. (*c*) Quantification of F-actin staining in the cell cortex does not suggest any changes in SLMAP3^−/−^ MEFs. (*d*) Depletion of SLMAP3, STRN3 or both have very little impact in focal adhesion of MEFs, as increase in number or size of focal adhesion in these cells were not observed. Images scale bar = 8 μm. Averages from multiple fields of each of three biological replicates plotted. **p < 0.01, ***p < 0.001.

SLMAP3^−/−^ MEFs presented subtle change in cell morphology observable in phase contrast microscope, with rounder and smaller appearance. To examine this further, we stained cells for F-actin with phalloidin and measured their dimensions with the parameters area, eccentricity, major axis length, minor axis length and perimeter and some of them were significantly different in SLMAP3^−/−^ MEFs versus WT (figure 5*a*). Since SLMAP3 localizes at the nuclear envelope [4–6], we also assessed any changes in nuclear dimension, but none of the parameters reached statistical significance in SLMAP3^−/−^ MEFs (figure 5*b*).

Cytoskeleton regulation has been associated with multiple STRIPAK members [33,105–112]. One of the mechanisms involves the regulation of ERM proteins, known to control F-actin in the cell cortex. The kinases MST3/4 and MAP4K4 were demonstrated to induce the phosphorylation and activation of ERM proteins [106,110,112,113], whereas STRN3 and STRIP1 negatively regulate MST3/4 in this process [106]. Given the association of SLMAP3 with STRIPAK and the changes in morphology of SLMAP3 deficient MEFs, we measured the F-actin staining in the cell cortex, but no statistical differences were found compared to WT cells (figure 5*c*).

The second mechanism proposed for the STRIPAK members to affect cell morphology by focal adhesion (FA) regulation [33,109,111,112]. MAP4K4 was shown to be involved with FA disassembly [111,112]; STRIP1 deficient MEFs exhibited reduced number of FA with increase in their clustering [33]; and depletion of CCM3 in cancer-associated fibroblasts increased the size and number of FA per cell [109]. To assess if FA was affected in SLMAP3^−/−^, we stained cells for paxillin. We also included in this analysis STRN3 deficient MEFs generated with shRNA (electronic supplementary material, figure S2B). Although both SLMAP3^−/−^ and STRN3 depleted MEFs had significantly reduced area, we could not find statistical difference between focal adhesion density in the cells, or changes in the focal adhesion dimension, such as area, major axis length, minor axis length and perimeter (figure 5*d*). These results suggest that SLMAP3 acts differently from other STRIPAK members in regulating cell morphology through mechanisms independent of increased F-actin in the cell cortex or alterations in focal adhesions.

### 2.4. Impact of SLMAP3 on migration and orientation of MEFs

**Figure 6.**
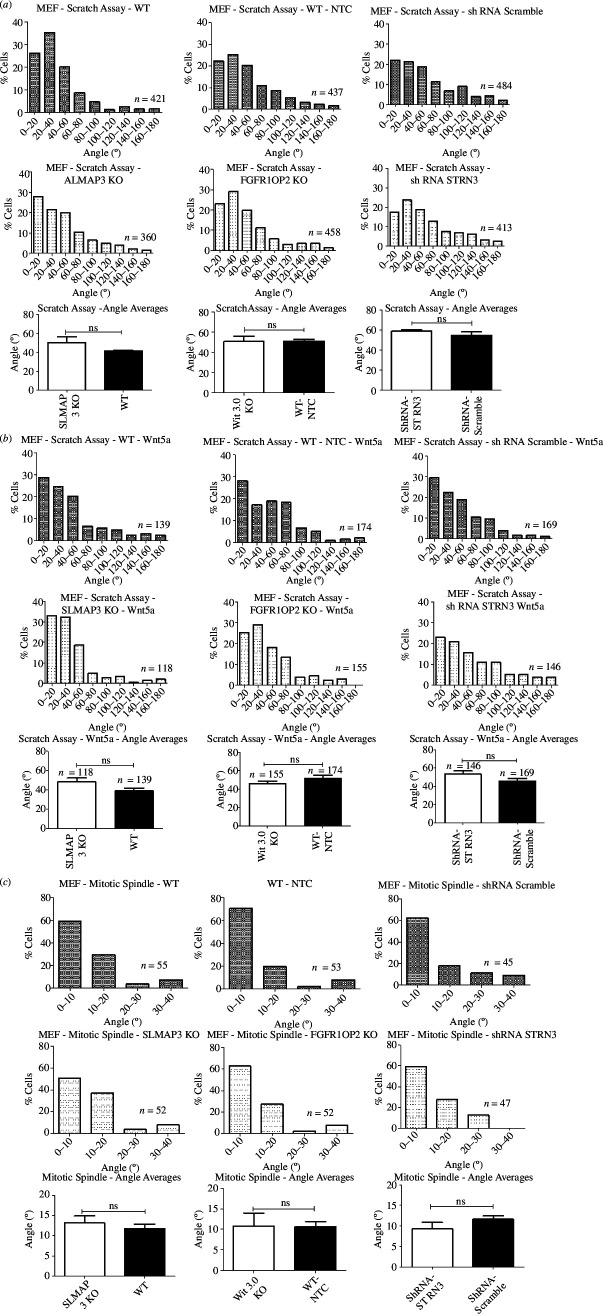
SLMAP3^−/−^ MEFs do not present orientation defects. (*a*) Cell orientation of migratory cells in a scratch assay was measured for MEFs with depletion of either SLMAP3, FGFR1OP2 or STRN3, but the average migration angles were similar to their corresponding controls, (*b*) even when cells were treated with Wnt5a. (*c*) Quantification of mitotic spindle angle does not suggest orientation defects on MEFs with depletion of either SLMAP3, FGFR1OP2 or STRN3, with average angles without statistical significance. For all analyses, the number of cells considered for the histogram (across three biological replicates) is indicated in the images. For each histogram, we plotted the averages of each of the three biological replicates (angle averages plots), therefore *n* = 3. Exception is (*b*), in which we plotted all cells together in the angle average plot. ns, not significant.

Polarized cell migration is known to be affected by PCP [82–86], and we examined if the loss of SLMAP3 in MEFs would lead to any polarity defects. Immortalized SLMAP3^−/−^ MEFs, which also lack SLMAP2, were first starved for 24 h, followed by scratch and migration for 6 h with or without the PCP ligand Wnt5a [57]. Cells of only the first or second layers in the migration edge were considered, and the angles were measured in individual cells in reference to their centrosome and Golgi localization, found in the front of the nucleus towards the migration edge [114]. We also generated and examined FGFR1OP2 and STRN3 depleted MEFs for comparison because of the lethality caused in mice with the knockout of either of these STRIPAK components [31,37] is similar to that observed in SLMAP3^−/−^ embryos. Although we found a shRNA that could deplete STRN3 expression (electronic supplementary material, figure S2B), the four shRNAs designed to target FGFR1OP2 could not reduce its expression (electronic supplementary material, figure S2C). Then, we designed two sgRNAs for CRISPR/Cas9 mediated knockout (electronic supplementary material, figure S2D,E), and we obtained a FGFR1OP2 knockout colony with sgRNA723# (colony 12) (electronic supplementary material, figure S2E), which was used for the experiments. The analysis with these generated cells indicated no changes in the angle of orientation, with the angles varying between 0˚ and 60˚ in all genotype types (figure 6*a*), even after treatment of the cells with Wnt5a (figure 6*b*).

We assessed the mitotic spindle orientation because it is known to be regulated by PCP as well [87–91]. Changes in this angle may also lead to the formation of the polycystic kidney, where disoriented division of the epithelial cells from the nephron tubules results in the enlargement of the diameter of these structures and reduced lengthening [115–117]. The mitotic spindle is also affected in MEFs lacking pericentrin [92], centrosomal protein that we identified as an interactor of SLMAP3 [6]. Mitotic spindle orientation was assessed by staining for γ-tubulin as a centrosome marker and acquisition of images in Z-stack. SLMAP3, FGFR1OP2 and STRN3 depleted MEFs were examined and the coordinates of the centrosomes were used for the arctan function to obtain the angle of the mitotic spindle, as described [92]. Although we observed a subtle reduction in the mitotic spindle angles in SLMAP3 KO MEFs, the angles in FGFR1OP2 KO MEFs, cells with drastically reduced SLMAP3 expression, was similar to that in control cells, so we attributed the changes to experimental variability (figure 6*c*).

**Figure 7.**
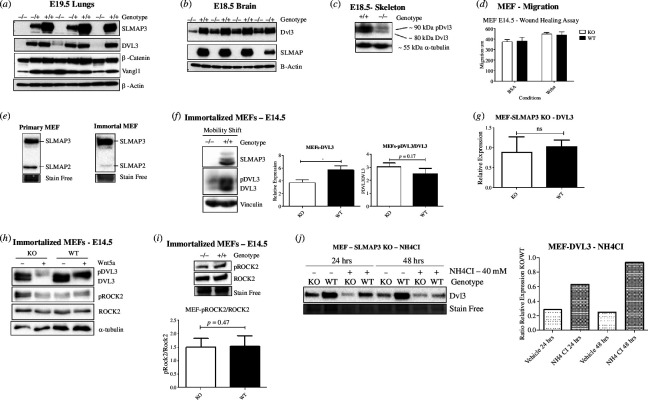
SLMAP3^−/−^ MEFs do not present defects in PCP or migration. (*a*) E20.5 lungs, (*b*) E19.5 brains and (***c***) E18.5 skeleton lacking SLMAP3 display reduced expression of DVL3. (*d*) Primary MEFs do not display migration defects. After 16 h, the migration of SLMAP3^−/−^ MEFs was comparable to the WT cells (*n* = 9). (*e*) Differently from primary MEFs, immortalized cells have almost no expression of SLMAP2. (*f*) DVL3 expression is reduced in immortalized cells, although its activation is not different from what is seen in WT MEFs (*n* = 3). (*g*) DVL3 transcripts are not reduced in cells lacking SLMAP3, as indicated by our RT-qPCR analysis (*n* = 3). (*h*) DVL3 in immortalized SLMAP3^−/−^ MEFs is still responsive to Wnt5a. (*i*) Immortalized MEFs do not have changes in ROCK2 activation status. *n* = 3. (*j*) With NH_4_Cl treatment the DVL3 protein levels in SLMAP3^−/−^ MEFs is comparable to WT cells. *p < 0.05.

We further investigated the PCP pathway and noted a significant reduction in the expression of DVL3 protein in the affected tissues, including lungs (figure 7*a*), brain (figure 7*b*) and skeleton (figure 7*c*). Surprisingly, DVL3 expression was not reduced in SLMAP3^−/−^ MEFs (electronic supplementary material, figure S3A), nor was its activation affected upon Wnt5a treatment (electronic supplementary material, figure S3B). Expression of the PCP-associated proteins including MUSK1, RhoA (electronic supplementary material, figure S3C) and ROR2 (electronic supplementary material, figure S3DE) was not impacted. Phosphorylation and activation of ROCK2 and JNK1/2 were not affected in SLMAP3^−/−^ MEFs either, even after Wnt5a treatment (electronic supplementary material, figure S3F). The migration of MEFs after 16 h, with or without Wnt5a treatment, did not indicate any changes (figure 7*d*).

As no changes in DVL3 protein levels were seen in MEFs despite changes observed in SLMAP3^−/−^ embryos, we analysed DVL3 expression after the immortalization of MEFs. Primary MEFs from E14.5 embryos were found to still express the approx. 45 KDa SLMAP2 isoform, which is lost upon their immortalization (figure 7*e*). We hypothesized that SLMAP2 may be compensating in primary SLMAP3^–/−^ MEFs to maintain DVL3 protein levels. Analysis after immortalization of primary SLMAP3^–/−^ MEFs indicates a dramatic decrease in DVL3 protein levels (figure 7*f*), with no changes in transcripts (figure 7*g*). However, even with reduced DVL3 expression, its activation was not altered (figure 7*f*), nor was its response to Wnt5a (figure 7*h*). ROCK2 activation and protein levels did not change either (figure 7*h*,*i*).

DVL3 protein can be degraded by both lysosomal and proteasomal mechanisms [118–120], and we examined if SLMAP was responsible for its stability by regulating these pathways. MEFs were treated with MG132, a proteasome inhibitor [121], or NH_4_Cl, a lysosomal degradation pathway inhibitor [122,123]. NH_4_Cl treatment was found to restore DVL3 protein levels in SLMAP3 deficient MEFs to that seen in WT cells after 48 h (figure 7*j*) while MG132 was without any effect on DVL3 after 24 h (electronic supplementary material, figure S3G). These results demonstrate that SLMAP is important for regulating DVL3 protein levels specifically by preventing its degradation by the lysosome. Thus, MEFs lacking SLMAP3 do not have orientation deficits in our experimental conditions, despite the reduced expression of DVL3, which was also notable in SLMAP3^–/−^ embryos and may contribute to the phenotypes observed.

### 2.5. The knockout of SLMAP3 or FGFR1OP2/Wit3.0 results in defective primary cilia formation

**Figure 8.**
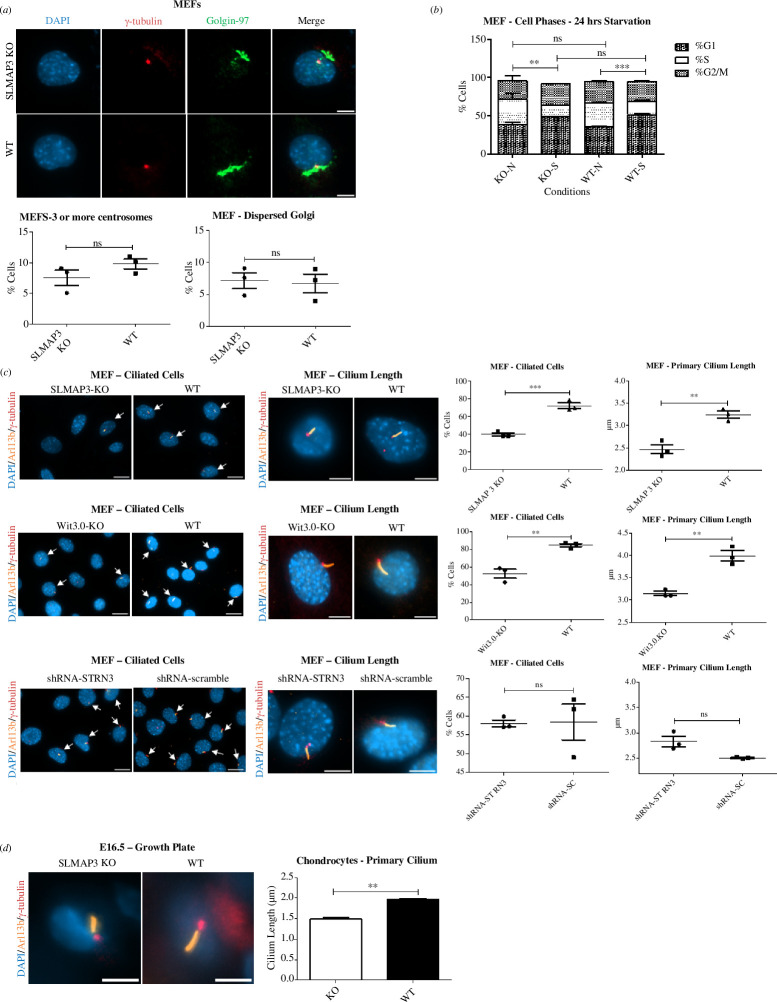
SLMAP3 and FGFR1OP2/Wit3.0 impact primary cilia formation. (*a*) Loss of SLMAP3 in MEFs does not cause centrosome or Golgi abnormalities. Averages of multiple fields of each of three biological replicates plotted, therefore *n* = 3. (*b*) Starvation of both SLMAP3^–/−^ and WT MEFs for 24 h with medium containing 0.1% FBS (*s*) enriched cells in G1 phase when compared with cells treated with 10% FBS medium (*n*). *n* = 3. Statistical significance is shown for %G1. (*c*) Knockout of either SLMAP3 or FGFR1OP2 reduced the number of ciliated cells and cilium length, which was not observed in MEFs with depleted STRN3. Arrows indicate ciliated cells. Averages of multiple fields of each of three biological replicates plotted, therefore *n* = 3. (*d*) Reduced cilium length was also observed in chondrocytes from SLMAP3^–/−^ growth plates. Averages of multiple fields of each of three biological replicates plotted, therefore *n* = 3. For (*a*), (*c*) and (*d*), single-cell images, scale bar is 8 μm; for images with multiple cells, scale bar is 20 μm. **p < 0.01, ***p < 0.001.

Given the associations of SLMAP3 with the centrosome [4,6,9], it is plausible that the lack of SLMAP3 could cause disturbances in this structure and result in the phenotypes observed in SLMAP3^–/−^ mice. However, the orientation assays indicated that during migration and cell division, the centrosomes are properly localized (figure 6*a, b* and *c*). Depletion of SLMAP3 in HeLa was reported to increase the number of centrosomes per cell [9]; however, no changes in centrosome numbers were found in MEFs depleted of SLMAP3 (figure 8*a*). Considering the association between centrosome and Golgi, we also assessed this organelle. Studies showed that depletion of CCM3, STK24 and MST4 induces the dispersal of Golgi apparatus [113,124], suggesting a role of STRIPAK in this organelle. However, we did not detect any changes in the Golgi in SLMAP3^–/−^ MEFs (figure 8*a*). In microtubule nucleation assays, we found that SLMAP3 was not important for the microtubule nucleation activity either [6].

The mother centriole from the centrosome gives rise to the basal body of the primary cilium, and mutations in associated proteins result in birth anomalies with systemic manifestation [69,125]. Disturbances in primary cilium could potentially explain the phenotypes observed in SLMAP3^–/−^ embryos. To analyse this structure in MEFs lacking SLMAP3, we first examined if they could undergo cell cycle arrest after 24 h of starvation to allow primary cilium growth. Both MEFs and C2C12 lacking SLMAP3 have reduced expression of the cyclin/CDK inhibitor CDKN1A (electronic supplementary material, figure S1C) and that could impair cell cycle arrest. However, SLMAP3^–/−^ MEF arrested in G1 phase as seen for WT cells (figure 8*b*). Next, we aimed to analyse primary cilium not only in SLMAP3^–/−^ but also in FGFR1OP2 and STRN3 depleted cells, given their partnership with SLMAP3 in the STRIPAK. After 24 h of starvation, we found that SLMAP3 or FGFR1OP2 knockout MEFs had reduced number of ciliated cells and cilium length, but not STRN3 deficient cells (figure 8*c*). The reduced cilium length was also observed in SLMAP3^–/−^ chondrocytes of humerus growth plate (figure 8*d*). These results suggest that both SLMAP3 and FGFR1OP2 are important in the formation of primary cilium, which may account for the similar phenotypes observed in mice with their respective knockout.

### 2.6. SLMAP3 is important for the protein stability of the STRIPAK members SIKE1 and FGFR1OP2

**Figure 9.**
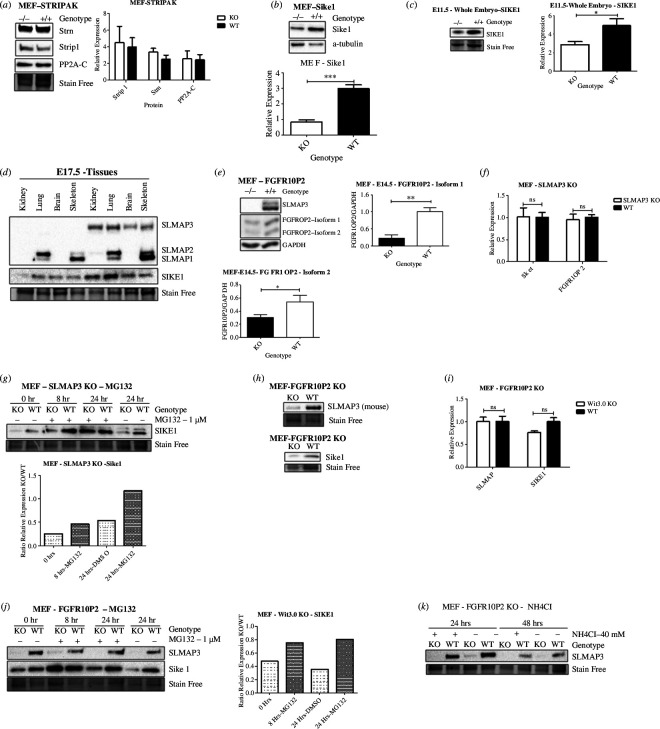
SLMAP3 impacts protein stability of SIKE1 and FGFR1OP2. (*a*) Loss of SLMAP3 does not affect the protein levels of STRIP1, Strn and the catalytic subunit of PP2A (PP2A-C) (*n* = 3). (*b*) Knockout of SLMAP3 leads to reduced protein levels of SIKE1 in MEF (*n* = 3), (*c*) whole embryos (*n* = 3) and (*d*) in multiple tissue. (*e*) The protein levels of FGFR1OP2 is also reduced in SLMAP3**^-/-^** MEFs (*n* = 3). (*f*) Transcripts of SIKE1 and FGFR1OP2, assessed by RT-qPCR, are not significantly altered in cells deficient of SLMAP (*n* = 3). (*g*) Treatment of SLMAP3**^-/-^** MEFs with MG132 was enough to increase SIKE1 expression after 24 h. (*h*) The knockout of FGFR1OP2 reduced protein levels of both SLMAP3 and SIKE1, but (***i***) did not change their transcripts, which was assessed by RT-qPCR (*n* = 3). (*j*) Treatment of MEFs deficient in FGFROP2 with MG132 increased the protein levels of SIKE1 but not SLMAP3. (*k*) The protein levels of SLMAP3 did not increase even with NH_4_Cl treatment. *p < 0.05, **p < 0.01, ***p < 0.001.

To gain insight into how the knockout of SLMAP3 could affect the STRIPAK complex, we analysed the protein expression of multiple STRIPAK members. No significant changes in expression of STRIP1, STRN and the catalytic subunit of PP2A were detected (figure 9*a*). However, we were particularly interested in the expression of SIKE1 and FGFR1OP2. SIKE1 was shown to recruit SLMAP3 to the STRIPAK by interacting with STRN3, and depletion of SIKE1 or SLMAP3 reduced the expression of each other in HGC-27 cells [39]. Decreased SIKE1 expression was notable in SLMAP3^–/−^ MEFs (figure 9*b*), whole embryos (figure 9*c*) and in different tissues (figure 9*d*). Although cells lacking SLMAP3 have reduced expression of SIKE1, the biological significance of this remains to be further addressed, since the knockout of SIKE1 in mouse does not result in any obvious phenotype [126]. Nevertheless, the knockout of FGFR1OP2 results in an astonishing resemblance to the phenotype of SLMAP3^–/−^ embryos, with lethality of 100% penetrance, shorter body size, reduced limbs and tails and abnormal face morphology [37]. Similarly to SIKE1, FGFR1OP2 expression was reduced in SLMAP3^−/−^ MEFs at protein level (figure 9*e*), with no changes in transcripts (figure 9*f*).

To examine if SLMAP3 affects SIKE1 stability through proteasomal mechanisms, we incubated cells with the proteasome inhibitor MG132 [121], and after 24 h of treatment, SIKE1 expression in SLMAP3^–/−^ MEFs was restored to levels seen in WT cells (figure 9*g*). These data imply that SLMAP3 protects SIKE1 from proteasome degradation. Next, we sought to examine the importance of FGFR1OP2 for SLMAP3 expression and observed that the expression of not only SLMAP3 but also SIKE1 protein was reduced in FGFR1OP2 KO MEFs (figure 9*h*), without changes in their transcripts (figure 9*i*). Treatment of these cells with MG132 lead to preservation SIKE1 while SLMAP3 protein was still absent even after 24 h of treatment (figure 9*j*). We also subjected these cells to NH_4_Cl, a known inhibitor of lysosomal degradation [122,123], and the level of SLMAP3 remained undetectable (figure 9*k*). This suggests that FGFR1OP2 regulates SLMAP3 protein levels by mechanisms other than the proteasome and lysosomal degradation.

## 3. Discussion

We report that loss of SLMAP3 is embryonic lethal in mice and stunts the development of multiple organs. Our data reveal that SLMAP3 may operate at multiple levels to guide development in a cell-/tissue-specific manner. The data indicate that SLMAP3 influences cell shape, growth and orientation, and operates through mechanisms involving the centrosome, cilia formation, protein stability of DVL3, and key STRIPAK components including SIKE and FGFR10P2. The discovery that SLMAP3 can selectively impact cilia formation in the growth plate *in vivo* and primary MEFs in culture define a unique mode of SLMAP action in this process.

Given the reports of the association of SLMAP3 with Hippo pathway, our first hypothesis was that the lack of SLMAP3 increased Hippo activity, resulting in smaller embryos. However, this is very unlikely to be the case since no changes in YAP phosphorylation or translocation were noted in SLMAP3 deficient cells. Furthermore, knockout of YAP in mouse results in embryonic lethality prior to organogenesis due to defects in placenta and in yolk sac vasculogenesis [127], while the knockout of both YAP and TAZ causes even a more severe phenotype, with lethality before embryonic implantation [128]. Previously, it was shown that depletion of SLMAP3 increases YAP phosphorylation and causes total exclusion from the nucleus in cell culture [46]; however, we never observed this in any tissues and cells analysed [6,97–99]. The knockout of SLMAP3 causing the complete absence of YAP in the nucleus should result in a phenotype at least similar to the complete ablation of YAP [127], which is not the case as SLMAP3^–/−^ animals can develop enough to be born and without observable placenta abnormalities. This highlights the importance of validation of cell culture findings with *in vivo* systems.

Detailed analysis of multiple organs of SLMAP3^–/−^ embryos implied that the PCP pathway could be responsible for the phenotypes observed. Similar defects with knockout of multiple PCP members are noted in lungs [52,53,61], skeleton [50,52,56], guts [59,60] and kidneys [49,55,61], as is the craniorachischisis [62,63] present in some of these embryos [99]. Furthermore, orientation defects were observed in the growth plate of SLMAP3^-/-^ embryos, a region known to be impacted by the PCP ligand Wnt5a [129]. In the case of kidney, nephronophthisis is a common manifestation of ciliopathies, and it is normally caused by mutations in NPHP genes, which encode for proteins from the transition zone of the cilia [125]. Knockout of VANLG2 and inversin/NPHP2 causes a similar type of polycystic kidney [49,55,61], highlighting the connections between PCP and cilia with a potential novel connection to SLMAP3.

Other investigations have also emphasized the relationship between cilia and Wnt signalling [77]. The depletion of ciliary proteins such as NPHP4, BBS4, BBS6, KIF3A, IFT88 and OFD1 was shown to positively influence the Wnt canonical pathway [74,75,78] while the overexpression of inversin/NHPH2 antagonized this effect [73]. Additionally, RPGRIP1L/NPHP8, BBS4, BBS6 and IFT88 were demonstrated to be required for the proper orientation and organization of stereociliary bundle of the cells from the organ of Corti [76,79,80]. Furthermore, Vangl2 was shown to genetically interact with BBS1 and BBS6 in mice and to localize in the cilium base of IMCD3 kidney cells [80]. Likewise, PCP proteins are required in determining ciliary patch orientation in ependymal cells, positioning the primary cilium in radial glial progenitor cells and organizing cell–cell polarity in both cell types [81]. Moreover, in *Xenopus laevis* mucociliary epithelium, DVL proteins regulate the docking of the basal body to the cell surface through a mechanism involving RhoA GTPase and contribute to the planar polarization of the basal body and directional cilia beating [130]. These studies support a role for PCP components and Wnt signalling pathways in primary cilium.

SLMAP3 is a centrosomal component [4] and serves a critical role in muscle development by guiding the formation of the MTOC in the nuclear envelope of differentiating myoblasts [6]. Given that depletion of SLMAP3 reduced the number of ciliated cells and cilium length and the similarities between SLMAP3^–/−^ embryos to that reported for models with PCP defects, it is plausible to think that the absence of SLMAP3 could disturb basal body functions. This contention is further supported by our finding that loss of SLMAP3 reduced DVL3 protein levels, which is similar to what is observed in aberrant expression of the ciliary proteins NPHP2 and NHPH4 that leads to enhanced degradation of DVL1, DVL2 and DVL3 [73,74], while suppression of BBS4 results in higher levels of DVL1 [75]. Conversely, a separate study found that depleting NPHP8 (Rpgrip1l), which reduces ciliated cell count and cilium length, is necessary for maintaining the stability and proper localization of DVL2 and DVL3 in the base of primary cilia in MDCK cells [76]. In MEFs, the knockout of NPHP2 increases DVL1 expression but depletes protein levels of DVL2/3 [84]. These studies exemplify the close relationship between DVL protein levels and the primary cilium and now point to SLMAP3 as a potential link between both.

Further credence to the role of SLMAP3 in cilia comes from interactome studies that detected not only SLMAP but also several STRIPAK members including STRN/3/4, STK26, STRK24, Mob4, MAP4K4 and STRIP1/2 in centrosome–cilium interface and centriolar satellites [131–133]. The SLMAP paralogues TRAF3IP3/T3JAM and CCDC136/NAG6 were also identified in these interactomes [132,133], and depletion of TRAF3IP3 was shown to reduce the number of ciliated RPE-1 cells [133]. Although FGFR1OP2 depleted MEFs had reduced number of ciliated cells and cilium length, similarly to SLMAP3^–/−^ cells, no changes were noted in STRN3-deficient MEFs. This suggest distinct roles for STRIPAK members in biology of cilia with SLMAP3 being prominent.

Considering the phenotypes observed in SLMAP3^–/−^ embryos, defective orientation is expected. Additionally, STRIPAK members STK25 and CCM3 were demonstrated to be required to polarized cell migration [113,124,134]. Depletion of FGFR1OP2 in rat fibroblasts impacted cell migration [42], and given its association with SLMAP3, the latter could have contributed to disorientation of cells to decrease migration. However, SLMAP3^–/−^ MEFs exhibited oriented migration and mitotic spindle angles comparable to the WT, implying that PCP defects observed in animals are not always reproducible as disorientation in cell culture. It is conceivable that SLMAP3 deficiency impacts cellular polarization in a cell-specific manner or that three-dimensional model systems might be required to reproduce the PCP defects observed *in vivo*.

Given the comparable phenotypes of SLMAP3 and FGFR1OP2 knockout mice and their association in the STRIPAK, one can envisage that they work together, with FGFR1OP2 being one of the most important adaptors for SLMAP3. Indeed, we found that SLMAP3 influences protein levels of both SIKE1 and FGFR1OP2, and in the case of SIKE1, SLMAP3 protects it from proteasomal degradation. Unfortunately, the mechanism of how FGFR1OP2 affects SLMAP3 protein levels remains elusive, but future investigations of degradation pathways with alternative compounds and tools [135] should clarify this. The finding that SLMAP3 protects SIKE1 from degradation is also intriguing. SIKE1 was reported to have protective roles in induced cardiac hypertrophy by repressing TBK1-AKT-mTOR cascade [136]. The specific knockout of SLMAP3 in cardiac progenitors resulted in smaller embryonic hearts but did not show significant changes in mTOR pathway activation [98]. Assessments of SLMAP3 cardiac protective roles with the induction of pathological hypertrophic program might reveal novel roles of this protein in cardiac physiology.

A key feature of SLMAP3^–/−^ MEFs was changes in cell morphology, with reduction in cell size and a shift towards a more rounded shape. However, no alteration in F-actin in the cell cortex and focal adhesions, which are regulated by STRIPAK [33,106,108–112], was detected. No changes in the activation of ROCK2 and JNK1/2, effector kinases of PCP known to regulate actin cytoskeleton [137,138], was observed. A more detailed investigation of F-actin cytoskeleton, including stress fibre measurements, analysis of the activation of the small GTPases RhoA, Rac or Cdc42, quantification of F- and G-actin and measurements of lamellipodia, might shed light into the mechanisms through which SLMAP3 orchestrates these cellular changes.

It is notable that SLMAP3 affects proliferation in a cell-specific manner since SLMAP3^–/−^ MEFs do not present aberrations in cell cycle dynamics, proliferation or cellular senescence, which were evident in C2C12 cells devoid of SLMAP3, similar to that observed in H9C2 cardiomyoblasts [98]. Further studies examining the impact of SLMAP on the cell cycle in different cell types will reveal which tissues it can impact. It will also clarify whether the reduced proliferation of chondrocytes in the growth plate of SLMAP3^–/−^ embryos is an autonomous or non-autonomous phenomenon, taking into account the widespread organ abnormalities.

Although RNA-seq analysis of E11.5 SLMAP3^–/−^ embryos indicated downregulation in genes associated with muscle development processes [6], interrogation by RPPA and RNAseq in SLMAP3-depleted MEFs did not reveal widespread changes. However, SLMAP3 loss did lead to a notable decrease in Arhgap36 transcripts in MEFs. Arhgap36 belongs to the Rho GTPase family and is involved in Gli transcription factors activation in the primary cilia [139]. How SLMAP3 may regulate this process needs consideration given the impact on cilia and is in line with our studies in muscle where it is crucial for guiding the MTOC and regulating gene expression in myogenesis [6].

**Figure 10.**
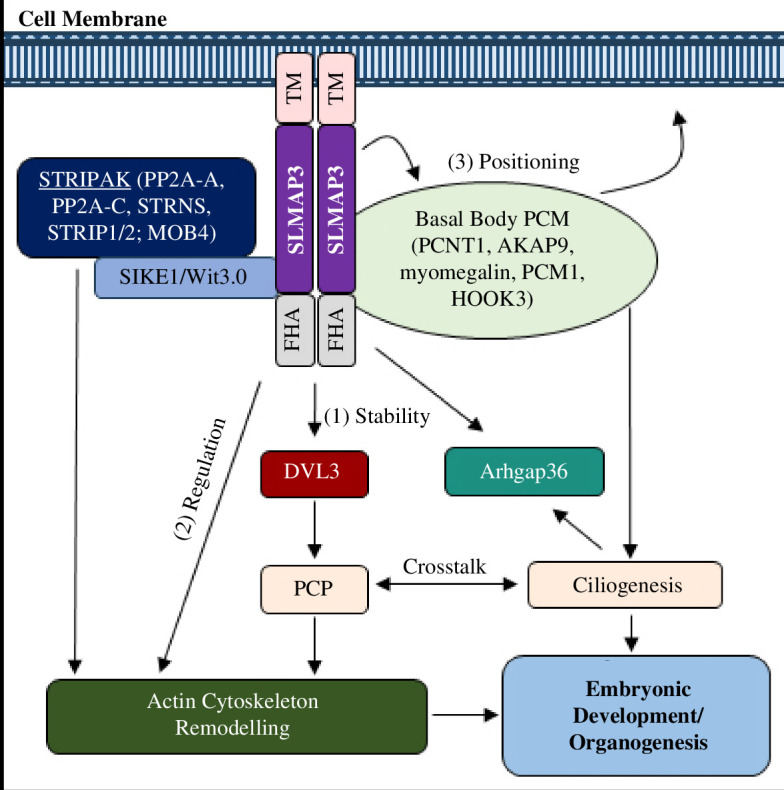
Proposed mechanisms of SLMAP3 for embryonic development and organogenesis. SLMAP3, a tail-anchored membrane, targets the centrosome to impact embryonic development and organogenesis by (1) protecting DVL3 from degradation for PCP signalling; (2) remodelling the actin cytoskeleton, a function that is shared with STRIPAK complex of which SLMAP3 is a component; (3) impacting primary cilia formation through positioning of basal body at the cell membrane by its novel associations with centrosomal (AKAP9, pericentrin, myomegalin) and centriolar satellite (PCM1 and HOOK3) proteins (6). Furthermore, SLMAP3 actions at the nuclear envelope could account for the gene expression of Arhgap36. FHA, forkhead-associated domain; TM, transmembrane domain; PCP, planar cell polarity; PCM, pericentriolar material.

Finally, the ubiquitous expression of SLMAP3 and the prevalent organ deficits observed in its absence implies that it is a crucial player in development. The data are revealing that SLMAP3 may serve diverse roles in a cell-specific manner that influence cell shape, growth and behaviour through mechanisms that involve the MTOC, cilia, PCP components and STRIPAK members. A model depicting the action of SLMAP3 is proposed (figure 10). As a member of STRIPAK, SLMAP3 could impact actin cytoskeleton, while its membrane association could help with docking of basal body at the plasma membrane by interactions with centrosome and centriolar satellite proteins, allowing the development of primary cilia. The ability of SLMAP to protect DVL3 against degradation would maintain PCP signalling, while its action at the nuclear envelope could account for gene regulation of Arhgap36. Given that SLMAP has no impact on YAP activity, the model here represents a logical segue into investigations on new mode of SLMAP3 action and its critical importance in organogenesis.

## 4. Conclusion

SLMAP3 is critical for development as its loss in mice results in widespread deficits in organogenesis with phenotypes reminiscent of defective PCP. Our data suggest that SLMAP3 modulates PCP at the level of primary cilium, a function that might be shared with its partner FGFR1OP2, with mechanisms involving protein stability of DVL3 and STRIPAK independent of Hippo signalling.

## 5. Material and methods

### 5.1. Transgenic mice generation

In this study, we used mice generated with the CMV driven Cre for the recombination of floxed SLMAP at exon 3 (reference transcript ENSMUST00000139075.8), as described elsewhere [6,97]. The Cre transgenic mouse was a gift from Dr Nemer Mona, University of Ottawa. Genotyping was performed following the procedures previously detailed [97].

### 5.2. Cell line generation and culture conditions

MEFs were isolated from E14.5 embryos as described [140]. For immortalization, MEFs were transfected with the plasmid SV40 1: pBSSVD2005 (a gift from David Ron; RRID: Addgene_21826) [141], following the protocol of the supplier. The immortalization consisted of at least five rounds of 1/10 splits of the transfected cells or until cellular senescence was no longer observed. Immortalized cells grew in culture for months without ever losing proliferation potential.

To generate MEF lines with depleted STRN3, we utilized shRNAs. As a control for the shRNAs, we employed a scramble sequence used before [97]. The shRNAs designed to target FGFR1OP2 failed to decrease its expression by more than 50%, and because of that we turned to CRISPR/Cas9. The sgRNAs for FGFR1OP2 are listed in the table below, and the Cas9 cleavage was confirmed with T7 endonuclease-based EnGen Mutation Detection Kit (New England Biolabs, cat. no. E3321S). For FGFR1OP2-sgRNA#2, we used the following pair of primers: 5′-GATGGCCTGCTCTAGACACT and 5′-TGGCGGTGAAGGATGTCTTA; for FGFR1OP2-sgRNA#723, we used the primers: 5′-TGAAAGTTGCCTGTAGTGGCT and 5′-AAACAAACATACAAGTGAGGCTAC. All these constructs were cloned into a lentivirus plasmid by Vectorbuilder. As a non-target control for CRISPR/Cas9, pLentiCRISPRv2 carrying a guide RNA targeting EGFP (a gift from Roland Friedel; RRID: Addgene_86153) (Addgene plasmid no. 86153) [142,143] was used, similarly to before [6]. The C2C12 myoblast cells utilized in this study, originally sourced from the American Type Culture Collection (ATCC), were previously engineered to possess a knockout of SLMAP3 via CRISPR/Cas9 technology, employing the pLentiCRISPRv2 vector (gift from Feng Zhang; RRID: Addgene_52961) [6,144,145]. All these constructs and target sequences are listed in table 1.

**Table 1.** Constructs and target sequences used in this study.

target	construct type	name	target sequence 5`- 3`
scramble	pLV-shRNA [146]	shRNA-SC	AGGATAAGCGTCAACGAATAGGTGA
STRN3	pLV-shRNA [147]	STRN3-shRNA#1	CACTGGTAGTGCGGTAATTTA
STRN3	pLV-shRNA [148]	STRN3-shRNA#2	AGCAAGGCAGACAGCTATTAA
FGFR1OP2	pLV-shRNA [149]	FGFR1OP2-shRNA#2	GAAAGATGACCCAGGTATAAT
FGFR1OP2	pLV-shRNA [150]	FGFR1OP2-shRNA_all	AGTCTGCCCTGGAACTGATAA
FGFR1OP2	pLV-shRNA [151]	FGFR1OP2-shRNA#1	GTCTATTGTGAAACGTTATTT
FGFR1OP2	pLV-shRNA [152]	FGFR1OP2-shRNA_custom#2	GTCTTACCTTCCTGTTCAG
FGFR1OP2	pLV-CRISPR-Cas9 [153]	FGFR1OP2-sgRNA#723	GCGAGTGGAAGCGATGAAGC
FGFR1OP2	pLV-CRISPR-Cas9 [154]	FGFR1OP2-sgRNA#2	TTCCTCCTGATACTAACGAA
SLMAP3	pLV-CRISPR-Cas9 [6]	SLMAP3-sgRNA#1	AGTCGGGCTTCCATACCATC
SLMAP3	pLV-CRISPR-Cas9 [6]	SLMAP3-sgRNA#2	ATACTCACTCTGAACGAAGT
EGFP	pLV-CRISPR-Cas9 [142]	sgRNA-NTC	GGGCGAGGAGCTGTTCACCG

We packed lentiviruses with Lenti-X 293 T cells, obtained from ATCC, using the psPAX2 and pMD2.G plasmids following Addgene protocol [155]. The lentivirus harvests were concentrated with Lenti-X-Concentrator (Clontech, cat. no. PT4421-2) and subsequently titred with PCR Lentivirus Titer Kit (abm no. LV900). Multiplicity of 20 was applied for transduction in MEFs, following the Addgene protocol [156]. MEFs transduced with shRNA-SC or sgRNA-NTC were subjected to puromycin selection at a concentration of 3 µg ml^−1^ for 3 days; cells transduced with STRN3-shRNA#1, STRN3-shRNA#2, FGFR1OP2-shRNA#2, FGFR1OP2-shRNA_all, FGFR1OP2-shRNA#1 and FGFR1OP2-shRNA_custom#2 underwent selection with G418 at 750 µg ml^−1^ for 6 days; and MEFs transduced with FGFR1OP2-sgRNA#723 and FGFR1OP2-sgRNA#2 were selected with blasticin at 1.5 µg ml^−1^ for 3 days. Depletion of STRN3 was confirmed by western blot. Expression of FGFR1OP2 was quantified in single-cell colonies generated by limiting dilution of the polyclonal cells transduced with sgRNA, according to Addgene protocol [157]. RT-qPCR was used for this quantification due to the technical challenges associated with FGFR1OP antibodies.

Cells were kept in humidified atmosphere with 5% CO_2_ and at 37°C. For MEFs, we used a medium containing high glucose DMEM (Wisent, cat. no. 319-005), 1 × MEM (Gibco, cat. no. 11140050), 20 mM HEPES, 10% fetal bovine serum (FBS) (Wisent, cat. no. 080-150), 1 × antibiotic–antimycotic (Gibco, cat. no. 15240062) and 0.1 mM β-mercaptoethanol, as described [158]. For C2C12 and Lenti-X cells, we used a medium containing DMEM (Wisent, cat. no. 319-005), 10% FBS (Wisent, cat. no. 080-150) and 1 × antibiotic–antimycotic (Gibco, cat. no. 15240062). Starvation medium consisted of DMEM (Wisent, cat. no. 319-005), 0.1% FBS (Wisent, cat. no. 080-150) and 1 × antibiotic–antimycotic (Gibco, cat. no. 15240062).

Regarding cell treatments, okadaic acid (Cell Signaling, cat. no. 5934) was used at 0.4 µM for 1 h and XMU-MP-1 (Selleckchem, cat. no. S8334) at 15 µM for 1 h. For PCP induction, MEFs were treated with 400 ng ml^−1^ of Wnt5a (R&D Systems, cat. no. 645-WN) for 2 h, or 16 h for migration assay and 6 h for the quantification of orientation of the migratory cells (scratch assay). Due to the availability of Wnt5a, for scratch assay, only one biological sample of each SLMAP3 KO, FGFR1OP2 KO and STRN3 KD and their controls was treated. For protein degradation studies, MEFs were treated with MG132 (Sigma, cat. no. 474790) at 1 µM for 8 and 24 h, and with NH_4_Cl at 40 mM for 24 and 48 h.

### 5.3. Lung explants

Lungs were isolated from E12.5 embryos for the explant growth. Dissection was done on cold PBS following the procedures described [159,160]. Dissected lungs were placed on autoclaved Whatman Nuclepore Track-Etched Membranes (Millipore Sigma, cat. no. WHA10417506), which was floating on a medium consisting of DMEM/F-12 containing l-glutamine, penicillin and streptomycin. The explants were kept in humidified atmosphere with 5% CO_2_ and at 37°C for up to 3 days. Images were acquired after isolation and every 24 h with the ThermoFisher EVOS FL Auto 2 system.

### 5.4. Histology

Tissues designated for histological analysis were fixed with 10% formalin. Subsequent procedures including embedding, paraffinization, sectioning and H&E staining were conducted by the Louise Pelletier Histology Core Facility at the University of Ottawa. For immunofluorescence studies, tissue deparaffinization, rehydration and antigen retrieval with citrate buffer pH 6.0 were performed according to an established protocol [161].

### 5.5. Alcian blue and Alizarin red

For general skeleton visualization, P0 mice were subjected to alizarin red and alcian blue staining of bones and cartilages, respectively, following an established protocol [162]. In brief, after euthanasia, the specimens were submerged for 30 s in 65°C to facilitate the removal of skin and organs. Subsequently, the samples were kept submerged overnight in each of the following solutions: 95% ethanol, acetone and then Alcian blue. The excess of staining was removed with washes with 70% ethanol, followed by another overnight incubation with 95% ethanol. In the following day, the samples were kept in 1% KOH for 1 h, followed by incubation with Alizarin red overnight. The excess and staining were eliminated with 50% glycerol : 50% (1%) KOH solution. For long-term storage, the specimens were kept in 100% glycerol. All the incubations were conducted at room temperature.

### 5.6. SDS-PAGE and western blot

Protein lysates from cells were extracted on ice by cell scraping and by homogenization of snap-frozen tissue. The lysis buffer consisted of 20 mM Tris pH 7.5, 150 mM NaCl, 1 mM EDTA, 1 mM EGTA and 1% Triton X-100. To each 10 ml of buffer, one tablet of Pierce Protease Inhibitor (Thermos Scientific, cat. no. A32955) and of Pierce Phosphatase Inhibitor (Thermo Scientific, cat. no. A32957) was added. For nuclear and cytoplasmic fractionation, we used a similar protocol described elsewhere [163]. In brief, cells were washed twice with cold PBS, followed by scrapping on ice with PBS containing 1 mM DTT and the Peirce Protease Inhibitor. The harvest was centrifuged at 1000*g* for 15 min at 4°C, followed by pellet resuspension with buffer A, consisting of 10 mM HEPES, 10 mM KCl, 1.5 mM MgCl_2_, 0.5 mM DTT and the Peirce Protease Inhibitor. After 15 min of incubation on ice, samples were handheld homogenized, and the cell lysis was verified with trypan blue staining of the nucleus every 20 strokes. The lysates were then centrifuged at 1000*g* for 5 min at 4°C, and the resulting supernatant was stored as cytoplasmic fraction. Using buffer A, the pellet was washed twice with centrifugation (1000*g* for 5 min at 4°C). After the final wash, the pellet was resuspended with the buffer A and homogenized with 20 strokes of a syringe attached to a 27 G needle, producing the final nuclear fraction. The denaturation of all protein lysates was achieved using SDS buffer containing 10% glycerol, 2% SDS, 62.5 mM Tris.HCl pH 6.8 and 10% β-mercaptoethanol and boiled for 5 min. Lysates were loaded on SDS-PAGE gel and posteriorly transferred to polyvinylidene fluoride membrane (Bio-Rad). Tris-buffered saline (TBS-T; 1 M Tris, 290 mM NaCl, 0.1% Tween 20 pH 7.4) was employed for membrane wash, blocking and antibody solutions. Antibody solutions were prepared with 5% milk in TBS-T or 5% BSA for primary antibodies targeting phosphorylated amino acid residues. Imaging was performed using the Bio-Rad ChemiDoc system, and densitometry analysis was conducted using Image Lab Software (Bio-Rad). Tables 2 and 3 list the antibodies utilized for western blot analysis.

**Table 2.** Primary antibodies used for western blot analysis.

primary antibodies				
target	company	catalogue	host	dilution WB
SLMAP	Novus Biologicals	NBP1-81397	rabbit	1:500
SLMAP	Novus Biologicals	NBP1-81398	rabbit	1:1000
STRN3/SG2NA (S68)	Novus Biologicals	NBP74572	mouse	1:500
MST1 (KRS2) (H-8)	Santa Cruz	SC-515051	mouse	1:200
MST2 (KRS1) (87 .K)	Santa Cruz	SC-130405	mouse	1:200
Phospho-MST1 (Thr183)/MST2 (Thr180) (E7U1D)	Cell Signaling	49 332	rabbit	1:500
YAP (1A12)	Cell Signaling	12 395	mouse	1:1000
Phospho-YAP (Ser127) (D9W2I)	Cell Signaling	13 008	rabbit	1:1000
Phospho-YAP(S397) (D1E7Y)	Cell Signaling	13 619	rabbit	1:1000
β-actin (AC-15)	Abcam	ab6276	mouse	1:2000
α-tubulin	Abcam	ab176560	rabbit	1:5000
GAPDH (GA1R)	Invitrogen	MA5-15738	mouse	1:5000
Lamin B1	Abcam	ab16048	rabbit	1:200
Vimentin (D21H3) XP	Cell Signaling	5741	rabbit	1:1000
Strn	BD Biosciences	6 10 838	mouse	1:1000
STRIP1/FAM40A	Bethyl Laboratories	A304-644A	rabbit	1:2500
PP2A-Cα/β (1D6)	Santa Cruz	SC-80665	mouse	1:200
SIKE1	Abcam	Ab121860	rabbit	1:200
FGFR1OP2	Novus Biologicals	NBP1-84148	rabbit	1:1000
DVL3	Cell Signaling	3218	rabbit	1:500
β-catenin (E247)	Abcam	ab32572	rabbit	1:8000
Vangl1 (D1J7X)	Cell Signaling	14 783	rabbit	1:1000
MUSK1	Invitrogen	PA1-1741	rabbit	1:500
RhoA (26C4)	Santa Cruz	sc-418	mouse	1:1000
ROR2 (D3B6F)	Cell Signaling	88 639	rabbit	1:1000
Cofilin (D3F9) XP	Cell Signaling	5175	rabbit	1:1000
Phospho-ROCK2 (S1366)	GeneTex	GTX122651	rabbit	1:750
ROCK2 (D-11)	Santa Cruz	SC-398519	mouse	1:200
Phospho-SAPK/JNK (Thr183/Tyr185) (81E11)	Cell Signaling	4668	rabbit	1:2000
JNK (D-2)	Santa Cruz	SC-7345	mouse	1:200
Vinculin (EPR8185)	Abcam	Ab129002	rabbit	1:5000

**Table 3.** Secondary antibodies used for western blot analysis.

secondary antibodies				
**target**	**company**	**catalogue**	**host**	**dilution WB**
Peroxidase AffiniPure Goat Anti-Rabbit IgG (H+L)	Jackson ImmunoResearch	111-035-144	goat	1:10 000
Peroxidase AffiniPure Goat Anti-Mouse IgG (H+L)	Jackson ImmunoResearch	115-035-146	goat	1:10 000

### 5.7. Reverse phase protein array

For the preparation of protein lysates for RPPA, primary MEFs isolated from E14.5 embryos were subjected to protein extraction with a buffer consisting of 1% Triton-X100, 50 mM HEPES pH 7.4, 150 mM NaCl, 1.5 mM MgCl_2_, 1 mM EGTA, 100 mM NaF, 10 mM Na_4_P_2_O_7_, 1 mM Na_3_VO_4_, 10% glycerol and one tablet of Pierce Protease Inhibitor (Thermos Scientific, cat. no. A32955) and of Pierce Phosphatase Inhibitor (Thermo Scientific, cat. no. A32957) to each 10 ml of the buffer. The extraction was carried out following the Functional Proteomics RPPA Core Facility procedures [104]. Proteins were denatured with one part of the 4 × SDS buffer containing 40% glycerol, 8% SDS, 0.25 M Tris-HCl pH 6.8 and 1/10 (volume/volume) β-mercaptoethanol and using three parts of the lysates. Samples were kept in −80°C until submission to the Functional Proteomics RPPA Core Facility.

At the facility, the lysates were diluted in five twofold serial dilutions in lysis buffer. Serially diluted lysates were arrayed on nitrocellulose-coated slides (Grace Bio-Labs) by the Quanterix (Aushon) 2470 Arrayer (Quanterix Corporation). A total of 5808 spots were arrayed on each slide including spots corresponding to serially diluted (a) standard lysates, and (b) positive and negative controls prepared from mixed cell lysates or dilution buffer, respectively. Each slide was probed with a validated primary antibody plus a biotin-conjugated secondary antibody. Antibody validation for RPPA is described in the RPPA Core website [104]. Signal detection was amplified using an Agilent GenPoint staining platform (Agilent Technologies) and visualized by DAB colourimetric reaction. The slides were scanned (Huron TissueScope, Huron Digital Pathology) and quantified using customized software (Array-Pro Analyzer, Media Cybernetics) to generate spot intensity.

Relative protein level for each sample was determined by RPPA SPACE, developed by MD Anderson Department of Bioinformatics and Computational Biology [164,165], by which each dilution curve was fitted with a logistic model. RPPA SPACE fits a single curve using all the samples (i.e. dilution series) on a slide with the signal intensity as the response variable and the dilution steps as the independent variable. The fitted curve is plotted with the signal intensities, both observed and fitted, on the *y*-axis and the log2 concentration of proteins on the *x*-axis for diagnostic purposes. The protein concentrations of each set of slides were then normalized for protein loading. Correction factor was calculated by (a) median-centring across samples of all antibody experiments and (b) median-centring across antibodies for each sample. Results were then normalized across RPPA sets by replicates-based normalization as described [166]. Details of the RPPA platform as performed by the RPPA Core are described by Siwak *et al*. [167].

### 5.8. Real-time quantitative polymerase chain reaction

The Primer-Blast tool (NIH) was used to design primers targeting all transcript isoforms of the gene of interest. Primers were validated by PCR product band size. RNeasy Mini Kit (Qiagen, cat. no. 74104) was employed for RNA extraction. For cDNA synthesis, we used iScript Reverse Transcription Supermix (Bio-Rad, cat. no.1708840). RT-qPCR was performed with the FastStart Universal SYBR Green Master (Rox) (Millipore Sigma, cat. no. 4913850001). GAPDH was used as the housekeeping gene. The samples were read on BioRad CFX 96 thermal cycler and data analysed on Bio-Rad CFX Maestro. The primers used are listed in table 4.

**Table 4.** Primers used in this study.

primers	forward (5′−3′)	reverse (5′−3′)
Arhgap36	AGAGACTGCTTACCACGAACTC	AAGCTCGTTCACCAGTACCTC
Cdkn1a	CCCGAGAACGGTGGAACTTT	AGAGTGCAAGACAGCGACAA
DVL3	AGTCAGCACAGTGAAGGCAGTCG	ATCAGCATCGGGGGACCATAGAGAG
SIKE1	GCTGTTCAGGTGGACGATAAC	AGCCACAGAGCTTTCTTTTCC
FGFR1OP2	CAAGCGAGTGGAAGCGATGA	GGGATGTCCGCAGTTCTTTG
SLMAP	AGATGTCATCCATGCTCCATTACC	GTATCTGAAGCCTCTTGGGTGATG
GAPDH	GGTTGTCTCCTGCGACTTCA	TGGTCCAGGGTTTCTTACTCC

### 5.9. RNA-sequencing

Total RNA was isolated from four SLMAP3^−/−^ and four wild-type E14.5 immortalized MEFs using the RNeasy Mini Kit (Qiagen, cat. no. 74104). Subsequently, the samples were submitted to the StemCore Laboratories Genomics Core Facility at University of Ottawa for library generation and RNA-sequencing as follows: total RNA was quantified using a Qubit (ThermoScientific) and its integrity was assessed on the 5200 Fragment Analyzer (Agilent Technologies). The libraries were constructed using Illumina Stranded mRNA Library Prep (Illumina). Following library QA/QC, the eight libraries were pooled on a NextSeq 2000, with the run module P2 100 cycles and approximately 400 M per run. RNA-seq post-processing and differential expression analysis were prepared by the Ottawa Bioinformatics Core Facility. Briefly, the reads were assigned to the GRCm38_GENCODE M25 transcriptome model using Salmon [168], and the differential expression of transcripts was analysed by DESeq2 [169]. The sequencing results have been deposited in NCBI Gene Expression Omnibus [170,171] and are accessible through GEO Series accession number GSE266611 (https://www.ncbi.nlm.nih.gov/geo/query/acc.cgi?acc=GSE266611). The data were validated by RT-qPCR, with the analysis of the expression of arhgap36 and cdkn1a.

### 5.10. Image acquisition and processing

Images were acquired at the Cell Biology and Image Acquisition Core Facility at the University of Ottawa. For the slides with H&E staining and cell plates, we used the ThermoFisher EVOS FL Auto 2 for image acquisition. For the cell migration, MEFs were incubated in the Incucyte ZOOM System (Sartorius) for 16 h, and images were analysed with the Scratch Wound Analysis Software Module for the measurements of wound width during the migration period. For the immunofluorescence experiments, we used the widefield microscopes as follows: images capturing cell migration orientation and mitotic spindle angles were acquired using the Zeiss AxioImager M2; the slides with anti-YAP/TAZ staining were analysed using Zeiss AxioObserver D1; the remaining immunofluorescent experiments were examined with Zeiss AxioObserver Z1. Slides for immunofluorescence were mounted using Vectashield Plus Antifade with DAPI (Vector Laboratories, cat. no. H-2000).

For quantification of YAP staining intensity in the nucleus, we used CellProlifer. First, we used the DAPI staining to segment the image and create the ‘nucleus’ object. The mean intensity of anti-YAP staining was measured inside these objects. We plotted the average of multiple fields of three biological replicates.

Chondrocyte orientation was quantified by determining the angle between the major axis of each chondrocyte and the growth plate axis, which we considered to be parallel to the different zones of the growth plate. Measurements were manually done with Fiji. For proliferation analysis in the proliferative zone of the growth plate, we counted the total number of cells in the field and compared it to the number of Ki67+ cells. In the kidney, we quantified the number of Ki67+ cells only in the epithelium of the proximal/distal tubules. These measurements were conducted across three biological replicates of SLMAP3^−/−^ and controls (wild type or SLMAP3^−/+^ that do not exhibit any phenotype).

The quantification of F-actin in the cell cortex was performed using cell objects generated with the segmentation of phalloidin staining. These objects were shrunk to 8 pixels (2.58 µm), and the band generated between the cells and the shrunk objects was considered the cell cortex, where phalloidin intensity was measured. For FA analysis, we used paxillin staining by applying the tool ‘enhance speckles’ on CellProfiler. Subsequently to image segmentation, we considered the parameters area, major axis length, minor axis length and perimeter for FAs. We excluded objects with either a major or minor axis smaller than 2.45 pixels (0.25 µm) because they fell outside the dimensions considered for FA [172]. For focal adhesion density, we considered the number of FA per area of cells.

In the migratory orientation assay, seeded cells were scratched and allowed to migrate for 6 h with or without Wnt5a. The angle of the cells was measured on Fiji between the overall migration axis (perpendicular to the scratch) and the individual cell migration axis (axis determined by the position of the Golgi and centrosome in front of the nucleus). For the quantification of mitotic spindle angle, MEFs were seeded at cell density of 8000 cell cm^−2^ and subjected to double thymidine treatment for cell cycle synchronization to increase the number of mitotic cells at the time of cell fixation. The treatment protocol consisted of 16 h with 10 mM thymidine, followed by 7 h of release. This was followed by another 16 h with 10 mM thymidine and subsequent 6 h of release, followed by immediate cell fixation and immunofluorescence. Images were acquired from the prepared slides by Z-stack, and the *x*, *y* and *z* coordinates of both centrosomes (*x*1, *y*1, *z*1 and *x*2, *y*2, *z*2) were acquired using the View5D from Fiji. The coordinates were applied in the arctan function, as previously described [92], that gives the mitotic spindle angle in radians using the equation below. The angles were converted to degrees and plotted. Imaris was used to validate this angle measurement method.


$$\theta \;\left(rad\right)=arctan\;\left(\frac{z2-z1}{\sqrt{{\left(x2-x1\right)}^{2}+{\left(y2-y1\right)}^{2}}}\right)$$

For all F-actin staining, we utilized Phalloidin-AF488 (Biotium, cat. no. 00042). The antibodies used for immunofluorescence are listed in tables 5 and 6.

**Table 5.** Primary antibodies used for immunofluorescence.

primary antibodies				
target	company	catalogue	host	dilution IF
YAP/TAZ (D24E4)	Cell Signaling	8418	rabbit	1:100
Ki67	Abcam	ab15580	rabbit	1:200
Paxillin	Abcam	ab32084	rabbit	1:100
Arl13b	Proteintech	17711–1-AP	rabbit	1:300
Golgin 97	Proteintech	12640–1-AP	rabbit	1:100
α-tubulin (DM1A)	Millipore Sigma	T6199	Mouse	1:300

**Table 6.** Secondary antibodies used for immunofluorescence.

secondary antibodies				
target	company	catalogue	host	dilution IF
Anti-Mouse IgG (H + L) Cross-Adsorbed Secondary Antibody, Alexa Fluor 647	Invitrogen	A−21235	goat	1:300
Anti-Rabbit IgG (H + L) Cross-Adsorbed Secondary Antibody, Alexa Fluor 555	Invitrogen	A−21428	goat	1:300
Anti-Rabbit IgG H&L, Alexa Fluor 488	Abcam	ab150077	goat	1:200

### 5.11. Flow cytometry

The flow cytometry readings were obtained at the Flow Cytometry & Virometry Core Facility at the University of Ottawa with the BD FACS Celesta system and processed on FlowJo software. For all analyses, doublet discrimination was done using the width and area for the forward scatter. Events gated from the plot with areas for forward and side scatter were used for quantification. Cell cycle analysis was conducted with DNA staining with PI according to the protocol by Moores Cancer Center, UC San Diego [173] and with the following details. After one wash with cold PBS, cells were resuspended with dropwise addition of 4 ml of 70% ethanol with concomitant gentle vortexing. Cells were maintained at 4°C for 24 h before further processing. PI staining was done by incubating cells at room temperature for 30 min and protected from light. Plots were obtained with the histogram of the linear PI intensity, and the cell cycle tool from FlowJo was used to gate the resulting plot in G1, S or G2/M phase. For apoptosis analysis, we used the Invitrogen kit (cat. no. V13242) containing PI and Annexin V conjugated to FITC following the protocol of the manufacturer. For positive death controls, cells were incubated with 1.6 mM H_2_O_2_ for 1 h. The events were plotted in log of FITC × PI intensity and gated in four quadrants according to the positive death controls. For SA-β-Gal assay with Cell Event Green Probe kit (Invitrogen, cat. no. C10850), we followed the protocol provided by the manufacturer. For positive control, we induced cellular senescence following the protocol previously described [174], treating cells with 600 µM of H_2_O_2_ for 2 h, and repeating the treatment in the following day. The induction of senescence was monitored by analysing changes in cell morphology in phase contrast microscope. The results were plotted as histograms of the log FITC intensity.

### 5.12. Statistical analysis

GraphPad Prism was used for all the statistical analyses. Data are represented by mean and standard error of mean. For single variable analyses comparing two groups, we employed *t*-tests. For analyses with three or more groups, we used one-way ANOVA followed by Newman–Keuls post-test. Analyses involving multiple variables were conducted using two-way ANOVA followed by Bonferroni post-test. Significance levels were denoted as * for *p* < 0.05, ** for *p* < 0.01, *** for *p* < 0.001, and ns for non-significant results.

## Data Availability

The RNA-seq results of MEFs from this study have been deposited in NCBI's Gene Expression Omnibus and are accessible through GEO Series accession number GSE266611 [175]. The RNA-seq data of E11.5 embryos from our previous study (6) were deposited in NCBI's Gene Expression Omnibus and are accessible through GEO Series accession number GSE230748 (https://www.ncbi.nlm.nih.gov/geo/query/acc.cgi?acc=GSE230748). The results obtained with RPPA, the clustering and heat map of relative protein expression, and uncropped images of all western blots of this study are available in the electronic supplementary material [176].
